# The Positive Influence of the Hidden Curriculum in Medical Education: A Scoping Review

**DOI:** 10.1007/s40670-025-02380-1

**Published:** 2025-04-11

**Authors:** Rhoda Meyer, Elize Archer, Liezl Smit

**Affiliations:** 1 Faculty of Medicine and Health Sciences, Department of Health Professions Education, University of Stellenbosch, Tygerberg, Parow, South Africa; 2 Faculty of Medicine and Health Sciences, Department of Paediatrics and Child Health, University of Stellenbosch, Tygerberg, Parow, South Africa

**Keywords:** Hidden curriculum, Medical education, Medical students, Faculty development

## Abstract

The negative influence of the hidden curriculum in medical education is often foregrounded in the literature. This scoping review explores the positive influence of the hidden curriculum on medical student’s learning, offering recommendations to harness its potential for teaching and learning. Following Arksey and O’Malley’s six-stage review process, four themes were developed: positive role-modelling, supportive team environments, positive institutional culture, and navigating ambiguity. These factors shape the positive influences of the hidden curriculum, influencing students’ implicit learning. By acknowledging the diverse aspects of the hidden curriculum, faculty and clinical teachers can prepare students to critically reflect on their learning experiences, recognizing the positive influences that enable learning and shape their identities as professionals.

## Introduction

The hidden curriculum has gained significant attention in educational discourse, as educators, researchers, and policymakers recognize its powerful influence on student learning [1]. While the formal curriculum is more explicit, the hidden curriculum operates beneath the surface, influencing attitudes, values, and behaviors, in ways often unnoticed yet highly significant [2, 3]. The concept of the “hidden curriculum” is widely ascribed to the work of sociologist Philip Jackson in the 1960 s [4]. Jackson defined the hidden curriculum as “the crowds, the praise, and the power that reinforce the dispositions of the students, regardless of what the teacher is working on at the moment” [4], highlighting that students learn implicit lessons from the social dynamics, routines, and power structures present in the learning environment. This early definition introduced the notion of an implicit, unwritten curriculum transmitted through the social and organizational structures of educational institutions. Building on the work of Jackson, other scholars further developed the concept of the hidden curriculum. For example, in his work on the relationship between social class and educational success, Bernstein highlighted how the hidden curriculum reflects and reinforces dominant cultural norms and values [5]. These contributions paved the way for further exploration and analysis of the concept of the hidden curriculum across various educational contexts.

In the context of medical education, the concept of the hidden curriculum has been explored by many who have sought to understand and describe it in the context of the classroom, simulation, or clinical environments [6, 7]. Students experience the hidden curriculum through exposure to the culture of the learning environment [7–9], where that which is learned may not be explicitly stated. The hidden curriculum may thus be viewed as cultural or institutional practices that are conveyed either formally or informally [10]. It includes implicit and often subtle behaviors, morals, and values which students learn from teachers and others across the various learning spaces in medical education [2, 8]. There is an understanding that these attributes are implicitly developed through role-modelling, where students acquire those critical skills and competencies necessary to function as healthcare professionals, like professionalism, communication, and empathy [11, 12]. Recognition of the importance of the hidden curriculum in medical education is therefore crucial, as it can support the efforts of curriculum developers to consciously align the hidden curriculum with explicit curricular goals, creating an environment that reinforces desired values, behaviors, and competencies essential for future doctors [3, 13].

The concept of the hidden curriculum is however multifaceted, and different perspectives contribute to its complexity, making it challenging to navigate. As mentioned earlier, the hidden curriculum is not something explicit or concrete [14], and often relates to the values and behaviors of individuals in a specific context, making it a challenge to objectively unravel and explore. There have been published studies on the hidden curriculum in medical education and its influence on learning; however, most studies focus on the negative aspects, failing to foreground the positive influence this curriculum can have on students’ learning. A lack of awareness of the hidden curriculum’s beneficial influences inherently limits the fullest realization of its potential for enriching students’ learning experiences. There is also a dearth of evidence on which aspects of the hidden curriculum would be most useful for medical students. In their scoping reviews, Raso et al. and Lawrence and colleagues [2, 15] echo this idea suggesting that there are far fewer insights depicting the hidden curriculum as a positive element within undergraduate health professions education.

The purpose of this scoping review, therefore, was to synthesize perspectives from previous studies related to the positive influences of the hidden curriculum on medical students’ learning. It was envisaged that an understanding of this will inform future research on how to better support faculty with harnessing the power of the hidden curriculum for teaching and learning. For this review, the following definition of the hidden curriculum by Gaufberg, Batalden, Sands, and Bell was used as a guide: “learning that occurs by means of informal interactions among students, faculty, and others [interpersonal–social] and/or learning that occurs through organisational, structural, and cultural influences intrinsic to training institutions” [7].

## Method

Since scoping reviews are useful for finding and charting relevant literature related to a specific topic, or concept under review [16], it was considered an appropriate approach for mapping the positive influence of the hidden curriculum across the medical education literature.

The objective of this review was to:

1. Synthesize the various views of the positive influence of the hidden curriculum on medical students’ learning in the health professions education (HPE) literature.

This scoping review was guided by the six stages for review proposed by Arksey and O’Malley [17] and adapted by Levac et al. [18], which include identifying the research question; identifying relevant studies; selecting the studies for inclusion; charting the data; collating, summarizing, and reporting results; and consultation.

The research team comprised faculty who teach on undergraduate and postgraduate HPE programs. Two members hold PhDs in HPE, while one member is near completion. This member also currently works as a senior clinician in a teaching hospital. The senior research assistant has an MPhil in HPE, as well as extensive experience in qualitative research and scoping reviews.

### Stage 1: Identifying the Research Question

The research question was developed in accordance with the Population-Concept-Context framework [19] as shown in Table 1. One broad question was decided on for the scoping review:

- What is the evidence of the positive influence of the hidden curriculum on medical students’ learning?

**Table 1:** Population-concept-context framework

Population	Health professions/medical education students
Concept	Hidden curriculum
Context	Learning

### Stage 2: Identifying Relevant Studies

We conducted our search across four databases, including, PubMed, Scopus, Web of Science, and ERIC. We chose not to limit the results by publication dates as the hidden curriculum represents a well-established theoretical framework in medical education. Including earlier literature allowed us to trace the evolution of this concept over time, informing current understandings of the positive influence of the hidden curriculum on medical students’ learning. The results were however limited to the English language so that the researchers could more accurately evaluate the literature without concerns about linguistic or cultural barriers impacting their understanding of the hidden curriculum concepts being discussed. Articles in peer-reviewed journals were included in the search and all study designs were considered, including quantitative, qualitative, and mixed methods. This review also included both empirical and secondary studies, e.g., reviews and guides. In consultation with a librarian, the following MeSH terms and keywords were selected to guide the review (Table 2).

**Table 2:** MeSH terms and keywords

MeSH terms	Keywords
Students, medical, HPE	Medical education or medical students
Curriculum	Hidden curriculum or informal curriculum
Learning	Learning and hidden curriculum

### Stage 3: Study Selection

**Fig. 1:**
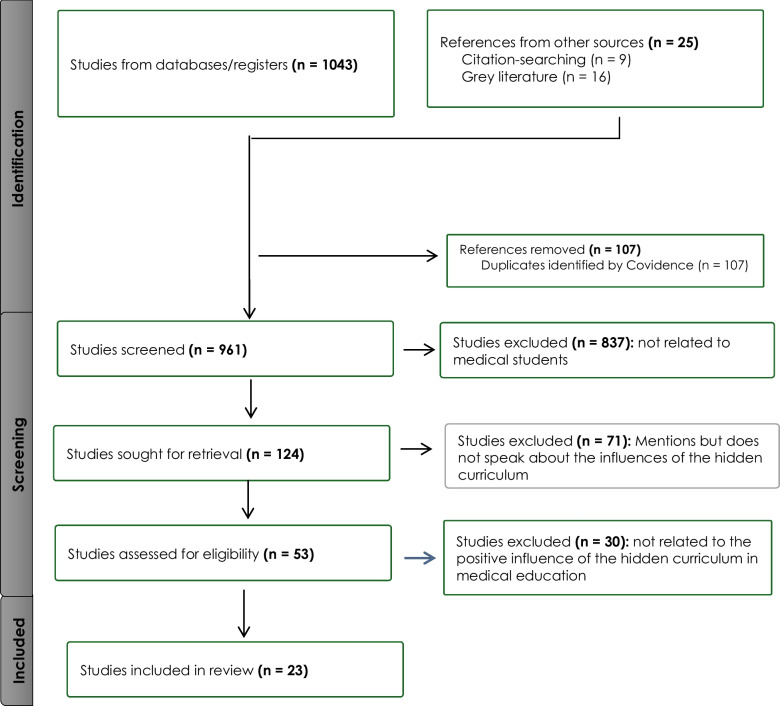
PRISMA chart showing the flow of studies

During the identification and study selection stages, all relevant studies which met the population, context, and concept criteria were uploaded onto a web-based software platform Covidence (https://www.covidence.org/), which is specifically developed to streamline the production of reviews. Relevant references gained through citation-searching and gray literature were also uploaded onto this platform. After all duplicates were removed, members of the research team reviewed the titles and abstracts. The selected abstracts were then screened by one reviewer and an independent senior researcher to identify relevant sources. We excluded studies that did not speak to the influence of the hidden curriculum on medical students’ learning. This was followed by retrieving and reviewing the full texts by all reviewers. While many of these studies defined the concept of the hidden curriculum, some of them focused primarily on the negative influences or outcomes of this curriculum, which did not align with the research question for this review, and were thus excluded. The research team then reviewed the final set of articles. The flow of studies included is presented in the (Fig. 1) below.

### Stage 4: Charting (Extracting) the Data

A document was created to guide the extraction of data from the full texts (Box 1). Data from these full texts was extracted independently by all three members of the research team by reviewing, extracting data, and charting relevant information. A senior research assistant was asked to independently conduct an additional check to ensure consistency. All data were then analyzed through content analysis, which was guided by the research question, keywords/ideas (Table 2), and earlier perspectives of Gaufberg, Batalden, Sands, and Bell [7].

An inductive approach was used. The first stage of the content analysis involved the research team familiarizing themselves with the content. Thereafter, similar concepts were grouped together to generate codes by assigning labels to segments of text that captured the key concepts, ideas, or findings relevant to the research question. Similar codes were then combined to form categories [20]. This process involved grouping related codes together, identifying patterns or relationships among them. Subsequently, a set of themes were constructed highlighting the various ways the hidden curriculum positively influences the learning experiences of medical students.

**Box 1.** Charting the data
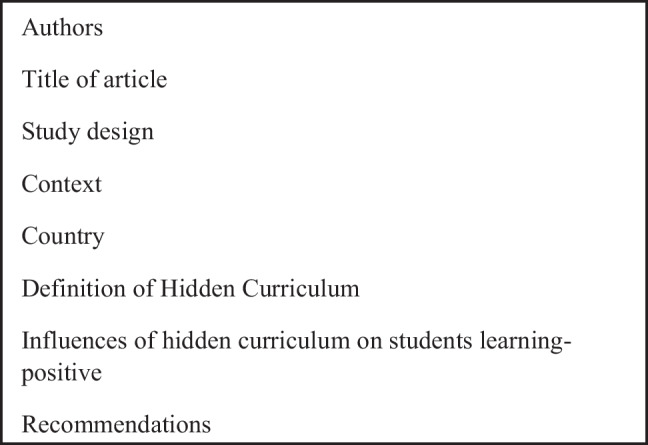


### Stage 5: Collating and Summarizing

Once the data had been analyzed, it was presented in the form of a table. Regular consultations were held by the team to discuss our interpretations of the text and to reach consensus on the analysis [20, 21].

### Stage 6: Consultations

Arksey and O’Malley suggest that a consultation with stakeholders regarding the findings of the scoping review is an optional step that could be included [17]. For this step, the research team shared the preliminary findings with stakeholders to understand if our findings resonated with their experiences of the hidden curriculum. Stakeholders were academic and clinical teachers of medical and other HPE programs in the faculty. All stakeholders offered valuable personal insights. Most agreed that our findings aligned with their experiences, offering suggestions in terms of how the hidden curriculum can be viewed. These suggestions were incorporated into the “Discussion” section.

## Results

**Fig. 2:**
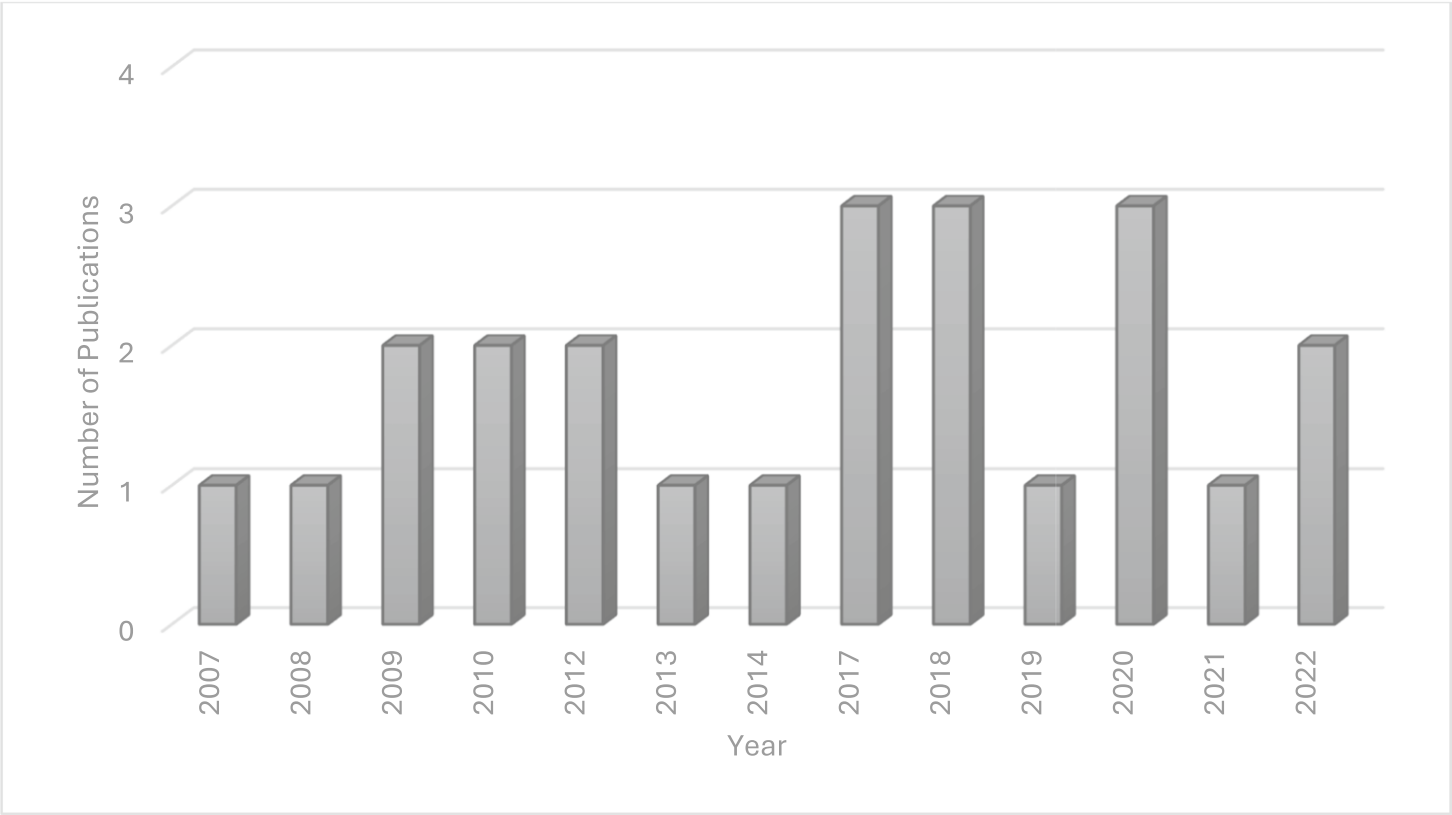
Number of publications per year highlighting the positive influence of the hidden curriculum on medical students’ learning

From the 1068 studies identified in the search, 107 duplicates were removed. Eventually only 124 studies which referred to aspects on the hidden curriculum in medical education were included. After assessing the studies for eligibility, 71 studies were removed because although they mentioned the term “hidden curriculum,” they did not speak to the influences of the hidden curriculum. In addition, 30 studies were excluded because they did not refer to the positive influences of the hidden curriculum in medical education, leaving 23 studies for full-text review. The years of publication ranged from 2007 to 2022, with the most being in 2017, 2018, and 2020 — see Fig. 2.

The types of articles included in this study were 18 empirical, four reviews, and an AMEE guide. The reviews explored the teaching of professionalism in medical education, the informal curriculum in family medicine, psychological safety in medicine, and an analysis of the main components of the hidden curriculum in medical education. For the empirical studies, the population included medical students, residents, and faculty. Through the process of content analysis, the following themes were constructed relating to the positive aspects of the hidden curriculum and its influence on medical students’ learning. These themes include positive role-modelling, supportive team environments, positive institutional culture, and navigating ambiguity (see Table 3 for a breakdown of the codes, categories, and themes).

**Table 3:** Codes, categories, and themes

Themes	Categories	Codes
Positive role modelling	Demonstration of professionalism Developing leadership Strengthens interpersonal and relationship-building skills	Fosters a sense of psychological safety Translates into positive student-patient behaviors Contribute to developing excellence and leadership qualities Learning about professionalism Recognition of dedication, persistence, and caring Shapes development of positive behaviors and professional values Acquisition of values and skills for professional growth Develops empathy and effective communication skills
Supportive team environments	Enhances positive learning experiences Promotes collaboration and teamwork abilities Fosters a growth mindset	Fosters collaborative practice Shapes development of working in a multidisciplinary working team Prepares students for teamwork Promotes positive interpersonal relationships Lead to motivation to help fellow students Develops values like respect, dignity, and supporting others Encouragement to learn and work harder Builds student confidence
Positive institutional culture	Facilitates acculturation and socialization into the medical community Fosters a commitment to lifelong learning Shaping professional role identity	Exposes students to organizational culture and survival strategies within that culture Students learn cultural competence A respect for cultural diversity enhances social interaction skills and tolerance Nurtures mutual respect Learning how to adapt to societal norms and values within the medical profession A sense of responsibility, duty, and service Helps students acquire skills for lifelong professional learning Learning about professional identity
Navigating ambiguity	Ethical and uncertainty Navigation Professional identity formation Cultivating empathy and tolerance	Students learn to deal with uncertainty, and ethical tensions Assists with identity formation of their roles as doctors Provides a space for contested reflection, which is crucial for professional identity formation Shapes students into agents of change Fosters a higher level of tolerance for others

### Theme 1: Positive Role-Modelling

Role-modelling as part of the hidden curriculum in medical education was a dominant trend in the articles reviewed [22–31]. These studies highlighted various effects of positive role-modelling on medical students’ learning, including the acquisition of values and skills necessary for professional growth [23] and the development of compassion, empathy, and effective communication skills [22, 24, 30, 32]. Some studies also highlighted key attributes and behaviors that characterize positive role models, including the display of behaviors related to dedication and persistence [27], the ability to foster a sense of psychological safety, and demonstrating excellence, leadership qualities, and proficiency in the field [24, 26, 29]. Additional attributes extracted from both empirical and review articles included demonstrating patient and student-centered behaviors such as listening, inspiring trust, politeness, and respect [25–27, 33]. In addition, the way students were treated by their role models influenced the way they interacted and responded to others; for example, Bandini et al. highlighted how students who were treated with compassion and respect extended these behaviors to patients and families [34]. Positive role models also have the potential to influence student identity, where students identify mainly with doctors who demonstrate positive relationships with their patients, as suggested by O’Sullivan et al. in their AMEE guide [35].

### Theme 2: Supportive Team Environments

Supportive team environments were associated with practices that fostered collaborative working relationships among members of healthcare teams with whom students interacted. In some studies, the team structure was seen as a form of support, which nurtured positive relationships with team members. Furthermore, being part of a team of different healthcare professionals provided an expanded understanding of the roles and responsibilities as a physician [27]. The learning-centeredness and supportive nature of the environments where students were placed, and the effectiveness of communication across the team were examples of factors which contributed to students’ positive learning experiences [36]. Being part of the team where members respond positively to students’ input also promotes a sense of psychological safety and develops student confidence [30]. Those students who were placed in cohesive teams seemed to have developed an increased tolerance to others [24].

In addition, it appeared that the hidden curriculum serves as an incubator for the development of relevant interpersonal skills which are critical for being a “good doctor” whose focus is on the quality of their relationship with their patients [37]. Collegial interactions between students and faculty, peers, and the healthcare team helped students to develop and acquire social interaction skills and other skills required for real-life situations [24]. Through positive interpersonal relationships, students developed innate attributes such as mutual respect, selflessness, and compassion [38]. Furthermore, while motivation is inherently intrapersonal, it appears that supportive team environments created conditions that enhanced students’ motivation to learn and fostered a growth mindset. The psychological safety experienced within cohesive teams enabled students to embrace challenges, and develop greater self-motivation, demonstrating how interpersonal dynamics can shape intrapersonal development. Studies also reported that supportive relationships promoted an increased sense of responsibility and the willingness to help fellow students [1, 24].

### Theme 3: Positive Institutional Culture

The hidden curriculum can be understood as an embodiment of the institution’s underlying culture and values. In the articles reviewed, a positive institutional culture represented the implicit messages, practices, and organizational culture that fosters a supportive, inclusive, and enriching learning experience for students. Some studies suggested that the hidden curriculum provides “richness” and depth in students’ learning and offers the opportunity to acquire the skills needed for lifelong learning [37]. Furthermore, placement in an environment that supports learning provides students with the confidence to seek assistance when needed as well as ways to access resources [27]. Acknowledging the role of students as learners also contributed to a positive learning experience creating a sense that teaching and learning, as well as students’ input, were valued [31]. Apart from the opportunity to develop competence, some students felt that they were being prepared to become agents of change, which created a feeling that others were willing to invest in their professional development [1].

In an attempt to create a positive institutional culture, some medical schools placed emphasis on certain attributes such as attendance and personal attire which seemed to influence students’ professional growth in a positive way [23]. In addition, positive institutional messages about the importance of certain aspects, such as unwavering duty, shaped how students valued this for their practice as healthcare professionals [1]. Linked to the institutional culture was the respect for cultural diversity demonstrated by the individuals in the institution. These practices helped students acquire the necessary social interaction skills to interact with diverse individuals and built a higher tolerance to others [24, 37]. While these studies spoke about a positive institutional culture, there were instances when the institutional culture was associated with negative learning experiences. However, it appeared that these were opportunities for students to develop an understanding of the institutional culture, and how to navigate this culture [37].

### Theme 4: Navigating Ambiguity

The positive influence of the hidden curriculum in medical education was also reflected in the practices and experiences that helped students develop the ability to manage uncertainty, complexity, and situations with conflicting messages. For example, Bandini et al. suggested that exposure to ethical-relational tensions helped students to navigate ambiguity present in the culture of the discipline [34]. Being exposed to ways to deal with uncertainty was also perceived as important to navigate the complexity within the clinical environment, as highlighted by Rothlind et al. (2020) in their review [39]. This complexity also provided a space for contested reflection, where students were guided through a process of reflection to explore their experiences, and the lessons learnt. This process, which is crucial for professional identity formation, also allowed students to identify the behaviors that they intended to adopt or avoid as part of their own professional conduct [40].

## Discussion

From the review, the hidden curriculum is perceived in different ways; what the positive influence of the hidden curriculum is for one context may mean something else for another. However, it must be noted that the intention of this review was not to provide a single perspective on the influence of the hidden curriculum, but to map the evidence available in the literature on the positive influence of the hidden curriculum on medical students’ learning.

**Fig. 3:**
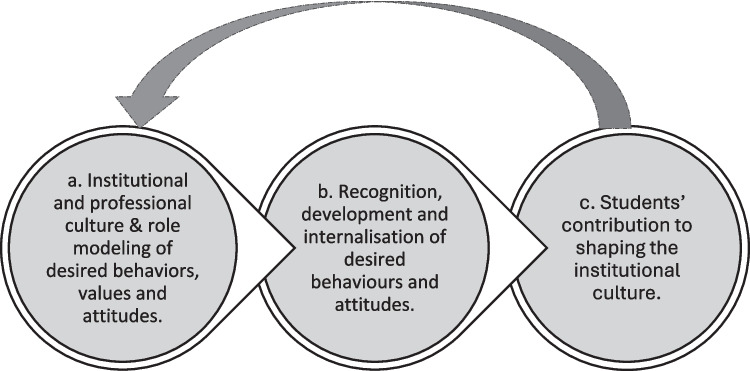
Facets of the positive influences of the hidden curriculum

This review offers three overarching ideas that became evident from the themes derived from the review of the articles (Fig. 3). Through further analysis and discussions among the team, we recognized that the four themes could be further synthesized into three broad, more conceptual overarching ideas that represent a higher level of analysis. This process moved our analysis from more descriptive themes to more interpretive concepts that assisted in explaining the relationships between the themes and how they influence each other in the context of the hidden curriculum. These include institutional culture and role modelling of desired behaviors, values, and attitudes; the recognition development and internalization of desired behaviors and attitudes; and students’ contribution to shaping the institutional culture.

The institutional and professional culture plays a fundamental role in shaping the hidden curriculum. If the institutional culture promotes values such as compassion, empathy, and effective communication skills, students are likely to adopt these values (Fig. 2a) [22, 34]. However, if the culture is marked by disrespect, or a focus on efficiency over patient-centered care, students may also internalize these attitudes and behaviors, potentially leading to various negative consequences, including poor patient outcomes and reduced patient satisfaction in their future practice [36, 41]. The professional culture of medicine also plays a role in shaping the hidden curriculum [37]. Trends and behaviors such as hierarchy among different healthcare professions, or biases towards certain patient groups, can send specific messages to students who may adopt these attitudes without being aware of it. In addition, the specific actions or behaviors role-modelled by faculty, clinical supervisors, or others in the learning environment can convey implicit messages about what is valued and what is expected of students as future doctors [27, 40]. Ultimately, these role models have an important effect on the development of professional identity of medical students as also suggested by Sarikhani et al. (2020) in their scoping review [29].

It is thus critical that faculty, clinical teachers, and staff in learning environments are acutely aware of these forces that shape the hidden curriculum, and actively try to influence it in a positive way [23, 42]. Greater awareness by faculty may also assist in creating opportunities for students to recognize certain behaviors and internalize these behaviors. Initiatives to influence the hidden curriculum may include open discussions about the implicit messages conveyed through institutional and professional culture, and encouraging students to critically reflect on, evaluate, and challenge harmful or outdated attitudes and behaviors [24, 34].

As mentioned, students eventually identify and adopt the implicit messages they receive regarding what is expected of them as doctors, with the danger that they could adopt undesirable behaviors (Fig. 2b). Thus, there needs to be efforts for students to formally recognize desired behaviors and attitudes. This can be done through establishing mentorship roles across the learning environment where guidance and feedback related to desired attributes can be provided from both clinical staff and faculty. Furthermore, opportunities to practice and internalize desired behaviors and attitudes can be built into clinical experiences, such as clinical rotations, interprofessional collaborations, and patient interactions [26]. In the classroom, activities that actively engage students in real-world scenarios can be used to develop a deeper understanding and appreciation for the relevance and application of these qualities [35, 38].

While the hidden curriculum is often shaped by institutional factors and role models, students themselves have the potential to support or even influence the institutional culture where learning and healthcare takes place (Fig. 2c). Through their actions, attitudes, and interactions, they can reinforce or challenge existing norms and practices [34], eventually shifting the implicit messages and expectations within the learning environment. Faculty and those in the clinical environment thus need to be prepared to recognize student voices, so that healthcare institutions can create a more collaborative and inclusive environment. In this way, students are empowered to contribute to the hidden curriculum and shape the culture that will ultimately influence their professional identities and practices.

## Implications for Faculty Development

Firstly, there needs to be an increased awareness of the hidden curriculum by faculty and clinical teachers. This can be facilitated through structured workshops where faculty get the opportunity to reflect on the hidden curriculum and its effect on teaching and learning [1]. By acknowledging the diverse aspects of the hidden curriculum, faculty and clinical teachers can prepare students to critically reflect on their learning experiences, exploring the ethical-relational tensions in the culture of the discipline [35] and recognizing the positive influences that enable learning as well as shape their identities as professionals [31]. Furthermore, curricula should be reviewed and renewed to ensure that aspects such as professionalism are enhanced.

Secondly, since teachers play a central role in shaping the hidden curriculum, faculty developers and managers need to create safe spaces for faculty and clinical teachers to reflect on their teaching practices, while encouraging discussions about implicit messages, values, and professional behaviors conveyed to students [26]. Faculty and clinical teachers should also be provided with feedback on their teaching practices, specifically related to how hidden messages impact student learning.

Finally, while it has been stated above that mentorship is necessary for students, it is clear that teachers also require mentorship. It is thus recommended that experienced faculty or clinical teachers are paired with newer colleagues. This mentorship relationship should include the transfer of tacit knowledge related to the hidden curriculum and how to maximize this for learning.

## Conclusion

This scoping review provides an overview of how the hidden curriculum is currently represented in the medical education literature, including the positive influences it has on student learning. This information holds significant relevance as the contexts in which medical students learn are often dynamic, providing unpredictable spaces for learning to occur. Through this review, we aimed to advance the conversation around the positive role that the hidden curriculum can play in enriching learning experiences in HPE.

By developing a deeper understanding of the hidden curriculum’s influence, we can inform our practices as faculty developers to assist teachers in maximizing its potential impact. When educators are aware of the implicit lessons conveyed through role modelling, hierarchies, and the informal culture of medical education, they can intentionally align these powerful forces with desired learning outcomes [10].

A better understanding of the hidden curriculum from the students’ perspectives provides faculty with invaluable insights into the contextual factors that influence learning. One area for further exploration is the positive influence of the hidden curriculum on teaching and learning from the perspective of faculty and clinical teachers. This awareness can guide efforts to tailor teaching approaches, shape learning environments, and address potential disconnects between the formal curriculum and the realities experienced in the learning environment [14].

## Data Availability

The data extracted for this scoping review were obtained through publicly accessible and subscription-based articles and databases. The datasets analyzed for this study are available from the corresponding author upon reasonable request.
